# Testing the Population‐Level Effects of Stress‐Induced Susceptibility in the Ranavirus–Wood Frog System

**DOI:** 10.1002/ece3.70728

**Published:** 2025-02-19

**Authors:** Jesse L. Brunner, Nicole C. Dahrouge, Erica J. Crespi, Tracy A. G. Rittenhouse

**Affiliations:** ^1^ School of Biological Sciences Washington State University Pullman Washington USA; ^2^ Department of Natural Resources and the Environment University of Connecticut Storrs Connecticut USA

**Keywords:** amphibian disease, epidemic, mesocosms, scaling, stress‐induced susceptibility

## Abstract

Chronic exposure to physical, chemical, and biotic stressors can make animals more susceptible to infections. Such stress‐induced susceptibility is widely expected to make disease—and epidemics—more likely and more severe. However, whether the impacts of stressors on individuals scale up to population‐level outcomes is uncertain, both theoretically and empirically. We initiated ranavirus epidemics in replicate mesocosm populations of larval wood frogs ( *Lithobates sylvaticus* ) exposed to conditions known to impact their individual susceptibility to ranavirus infections: low and high salinity crossed factorially with ambient or elevated temperatures. Contrary to expectations, epidemics were no more likely or more severe in stressful conditions, although increased temperatures did speed their progression. We discuss several potential reasons why the effects of stressors did not scale up to epidemics, but our results suggest caution in assuming the individual‐level effects of even well‐studied stressors necessarily scale simply to population‐level outcomes.

## Graphical abstract

Experimental ranavirus epidemics in populations of wood frog tadpoles were used to the hypothesis that individual‐level effects of stress and temperature predict population‐level outcomes. In contrast to conventional wisdom, environmental stressors did not appreciably change the outcome or severity of 96 replicate experimental epidemics in mesocosms.

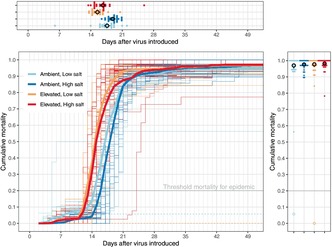

## 1 Introduction

Mass mortality events associated with infectious disease are on the rise (Fey et al. 2015), and stressors—challenging physical, chemical, and biotic conditions—are broadly thought to promote these events (Carey, Cohen, and Rollins‐Smith 1999; Hing et al. 2016; Kiesecker 2011). Chronic exposure to stressors is known to impact the physiological and immunological condition of animals, making them more susceptible to infections (i.e., the stress‐induced susceptibility hypothesis; Defolie, Merkling, and Fichtel 2020; Dhabhar 2014; Glaser and Kiecolt‐Glaser 2005; Hall et al. 2020; Rollins‐Smith 2017). Such stress‐induced susceptibility at the individual level, especially caused by anthropogenic stressors, is often implicitly or explicitly assumed to lead to more frequent and severe disease outbreaks in wildlife population (Brearley et al. 2013; Kiesecker 2011; Köhler and Triebskorn 2013). Indeed, researchers often find broad correlations between anthropogenic environmental influences and pathogen or disease prevalence (Brearley et al. 2013; James et al. 2015; St‐Amour et al. 2008) with the inference that these patterns were caused by the stressors. However, whether individual‐level effects of stressors scale up to cause worse population‐level outcomes depends in large part on how infections are changed (Lafferty and Kuris 1999; Lafferty and Holt 2003).

Theoretically, if stressors simply make hosts more easily infected, then stressors should increase the probability an introduction of infection leads to an epidemic and make the resulting epidemics larger and more rapid (Lafferty and Holt 2003), as is usually assumed. However, if stress instead (or also) leads to infected hosts being more likely to die with less intense infections or die faster (i.e., more virulent infections), then infected individuals may have less time or capacity to transmit their infections, in which case stressors might instead lead to smaller or slower‐growing epidemics (Lafferty and Holt 2003). These effects might also offset each other, leading to negligible effects of stressors on epidemic dynamics and outcomes. Thus, the distinct and possibly countervailing influences of stressors, or environmental challenges more broadly, on infections make it difficult to predict the population‐level consequences of chronic stress.

Empirical data illustrate these divergent population‐level outcomes. For instance, *Daphnia* exposed to toxic cyanobacteria are more susceptible to the gut parasite, *Caullerya mesnili*, and so the prevalence of *C. mesnili* was strongly correlated with cyanobacteria densities in a 12‐year time series (Tellenbach et al. 2016), suggesting this increased susceptibility contributed to *C. mesnili* epidemics. In contrast, while food stress made infections with the microsporidian gut parasite *Glugoides intestinalis* more lethal (and vice versa), populations with less food had reduced intensities and prevalence of infections in large part because host mortality regulated the parasite population (Pulkkinen and Ebert 2004). More broadly, numerous studies have shown that increases in exposure to parasite propagules that stem from increased foraging rates (e.g., in response to food stress) are more important for driving epidemics dynamics than individual‐level differences in susceptibility or infection intensities (Dallas, Hall, and Drake 2016; Dallas and Drake 2016; Hall et al. 2009; Shocket et al. 2019). Similarly, tadpoles subjected to stressors (e.g., pesticides and predators) are often more susceptible to trematode parasites, but their influence on infection prevalence is often complex and indirect (e.g., affecting densities of the snail intermediate host) as much or more than individual susceptibility (Rumschlag et al. 2019; but see Rohr et al. 2008).

Indeed, the strength of many of these studies—examining host–parasite interactions embedded in complex ecological systems with many interacting players—makes it difficult to evaluate the evidence for the population‐level consequences of stress‐induced susceptibility, per se. It is clear that stressors influence hosts and parasites (and vectors and intermediate hosts) through multiple pathways (Dallas and Drake 2014; Rohr et al. 2008), changing vital rates and densities (Gallagher et al. 2019; Pulkkinen and Ebert 2004; Rumschlag et al. 2019) or feeding rates and exposure (Civitello et al. 2013; Dallas and Drake 2014), as well as impacting host susceptibility. But while it is possible to statistically parse the relative importance of susceptibility relative to other factors (e.g., Rohr et al. 2008), there are few direct tests of how stress‐induced changes to host susceptibility, by itself, scale up to population‐level outcomes.

We set out to empirically test how the well‐documented individual‐level effects of two increasingly common and important environmental challenges—elevated salinity and temperature—scale up to population‐level dynamics and outcomes using the experimentally tractable wood frog ( *Lithobates sylvaticus* )–ranavirus system. Wood frog larvae are highly susceptible to ranavirus infections (Haislip et al. 2011), often experiencing mass mortality events in the wild (Brunner et al. 2021). As with amphibian declines more broadly (Carey, Cohen, and Rollins‐Smith 1999; Rollins‐Smith 2017), environmental stressors have been hypothesized as an important player in the emergence of these viruses (Brunner et al. 2015).

Both field and laboratory studies show wood frogs are physiologically sensitive to salinization, for instance, from runoff of road deicing salt, across multiple life history stages. Elevated salinity increases embryonic mortality, slows growth and development, and reduces activity of surviving larvae; has negative carry‐over effects on performance of juveniles; and increases bloating and corticosterone levels in adults (Brady 2013; Dahrouge and Rittenhouse 2022; Hall et al. 2017; Karraker, Gibbs, and Vonesh 2015; Lewis et al. 2021; Relyea et al. 2023). Elevated salinity also leads to more intense and lethal ranavirus infections in wood frog larvae following exposure, which has been associated with an augmented infection‐induced glucocorticoid hormone response, blunted immune activity (Hall et al. 2020), and a shift in gut microbiome assembly (Hughey et al. 2023). Moreover, vernal ponds near roads, where deicing salts are applied, are more likely to experience ranavirus‐related mortality events (Hall et al. 2020). Thus, in line with the stress‐induced susceptibility hypothesis, we predicted that these adverse effects of elevated exposure to road‐deicing salts on ranavirus infection would scale up to increase the probability and severity of ranavirus epidemics.

Elevated water temperatures—due to warming climates, reduced canopy cover, and earlier pond drying (Brooks 2009; Calhoun et al. 2017; Skelly, Bolden, and Freidenburg 2014)—are also likely to influence the dynamics and outcome of ranavirus infections (Ariel et al. 2009; Grant et al. 2003; Rojas et al. 2005; Speare and Smith 1992). Whether warmer temperatures lead to milder or more severe infections is difficult to predict because both viral replication (Bisht and Te Velthuis 2022; Granoff, Came, and Breeze 1966) and host responses (Herczeg et al. 2021; Ribas et al. 2009; Rollins‐Smith 2017) are temperature dependent and thus the outcomes may depend on a *mismatch* in thermal performance than temperature per se (Brunner et al. 2015; Cohen et al. 2019). Temperature also affects rates of feeding, growth, and development, all of which may influence susceptibility (Brunner et al. 2015). However, relatively small increases in water temperature (~2°C) can substantially increase the odds and speed of ranavirus‐induced mortality in wood frog tadpoles (Brand et al. 2016). Indeed, mortality events in wood frog populations in the wild are often associated with rising temperatures (Hall et al. 2018), although the threshold is rather warmer than that observed in the laboratory. In the end, we expected that elevated temperature would cause faster, if not necessarily more or less severe ranavirus epidemics, and might amplify the effects of elevated salinity on epidemics, as has been observed with direct toxicity (Green and Salice 2020).

To test our hypotheses, we tracked the course of experimentally induced ranavirus epidemics in 96 mesocosm populations of wood frog larvae, alongside 32 control mesocosm populations, assigned to one of four environmental treatments: ambient or +3°C elevated temperature crossed factorially with low‐or high‐salinity water. We then compared the proportion of introductions that caused epidemics, the cumulative mortality in the epidemics, the speed of the epidemics between treatments, and the severity of infections (viral load) across epidemics.

## 2 Materials and Methods

Thirty‐six partial *Lithobates sylvaticus* egg masses were collected from three wetlands within the University of Connecticut Forest (41°49′ N, 72°13′ W) on March 25, 2021 (Connecticut Department of Energy and Environmental Protection Scientific Collections Permit #1224001) and then maintained at ambient temperatures in 6‐quart plastic shoeboxes. Salinity was gradually increased (50–100 μs/day) in half of the shoeboxes over a 4‐week period to prevent mortality from rapid change in salinity, but so the early influences of elevated salinity were included in our study (Hall et al. 2017; Karraker, Gibbs, and Vonesh 2015). On April 14, 2021, when hatchlings reached a free‐swimming feeding stage (Gosner 1960 stage 25), larvae were haphazardly placed into groups of 40 in cups, mixing animals from different natal populations, and then a randomly selected group was added to each mesocosm. At this time, the temperature manipulations began.

Mesocosms consisted of 190‐L plastic cattle tanks filled with 150‐L well water, 10 large, dry oak (mixed *Quercus alba* , *Q. velutina* , and *Q. rubra* ) leaves, and 250 mL of water and zooplankton from a local wetland placed in an open field that received full sun and covered with 50% shade cloth (PAK Global, Cornelia, GA, USA). Water temperature in each elevated temperature mesocosm was raised to 3°C above that in a paired ambient temperature mesocosm, up to a maximum of 32°C, using a 300 W heater (GH300; Aquatop, Brea, CA, USA) controlled by an Arduino microcontroller (ArduinoNano ATmega328; Arduino AG, Cham, Switzerland). Salinity was elevated to 1900–2000 μS/cm—a moderately high level reflective of that in roadside ponds (Karraker, Gibbs, and Vonesh 2015)—by adding road salt (treated with calcium and tree lignin; DRVN Enterprises Inc., Rocky Hill, CT, USA) to the naturally low‐salinity (109–177 μS/cm) well water used in this study. Water in each mesocosm was circulated through a UV filter (Green Killing Machine 24 W; AA Aquarium, Pawleys Island, SC, USA) to clarify the water so that carcasses could be reliably detected, which also prevented infectious ranavirus particles from accumulating to unrealistic levels in the tanks (Brunner et al. 2017). Mesocosms were kept from overflowing during rains with drain holes at the same level in each mesocosm connected with vinyl tubing that drained to a buried 5‐gal bucket containing a CaO(Cl)_₂_ solution (Poolife TurboShock Shock Treatment, Innovative Water Care Global Corporation) to inactivate any ranavirus. Tadpoles in each mesocosm were fed 300 mg of mixed alfalfa pellets and tropical fish flakes twice weekly in April, and every other day beginning in May for the duration of the 52‐day experiment.

Three weeks after the mesocosms populations were constructed (~Gosner stage 26) and just prior to the start of the epidemics, populations were censused. Some small tadpoles had been entrapped in the UV filter intake and died, leaving populations varying in size from 32 to 40, with a mean of 39.3. Ranavirus transmission is not altered by tadpole density, especially over such a small range of densities (Brunner et al. 2017), so our study proceeded as planned. On May 3, 2021, ranavirus epidemics were initiated in 24 of the 32 mesocosms per environmental treatment with the addition of two ranavirus‐infected larvae (waterbath exposed to 2.5 × 10^4^ plaque‐forming units/mL of a local FV3 ranavirus isolate for the prior 24 h) that had been raised in the same conditions as their recipient mesocosm. In the eight remaining control mesocosms per treatment, two unexposed larvae were added as a sham inoculation. Introduced larvae were marked with a visible implanted elastomer (Northwest Marine Technology, Anacortes, WA, USA) so they could be distinguished from the susceptible residents and were excluded from analyses.

Mesocosms were checked daily for carcasses and metamorphosing individuals (= front limb emergence; Gosner stage 42) in a standardized fashion, looking under every leaf and behind the UV filter to ensure accurate counts. Metamorphosing animals were euthanized with 20% benzocaine gel applied to the ventral surface, both euthanized and dead animals were individually bagged and frozen for later screening. All metamorphs from ranavirus‐exposed mesocosms and 10 metamorphs from each control mesocosm were euthanized and tested for ranavirus; the remaining control animals were transferred to a separate study. This research was approved by the University of Connecticut Institutional Animal Care and Use Committee (Protocol #A20‐017).

Small pieces (≤ 2 mm^2^) of liver, interrenal gland, and intestines, as available, were dissected from the frozen carcasses. DNA was extracted from these combined tissues using Qiagen DNeasy blood and tissue kits, following the manufacturer's recommendations. All instruments were disinfected in a 50% solution of commercial bleach for ≥ 1 min and then rinsed in fresh water to prevent cross contamination. Pieces of liver from uninfected bullfrogs ( *Lithobates catesbeianus* ) or chickens were extracted with each set of samples as negative extraction controls. Extracted DNA was then run in triplicate 20‐μL quantitative real‐time PCR reactions targeting the viral major capsid protein (MCP) gene (Stilwell et al. 2018). Each 96‐well PCR plate included a serial dilution of known number of copies of a gBlock synthetic oligonucleotide (Integrated DNA Technologies, Coralville, IA, USA) containing the target sequence to serve as a standard and no‐template controls to detect contamination in the PCR setup. Samples with ambiguous results (e.g., one of three wells showing amplification) were re‐run. We used a threshold of five copies to separate positive from negative samples. Lower copy numbers would be consistent with low levels of sample contamination that might have occurred during sample collection, shipments, or processing, but would not be consistent with ranavirus‐induced mortality (Brunner et al. 2019; Brunner and Collins 2009).

We compared the proportions of animals dying (out of those recovered) or positive (out of those tested) among treatments using logistic regressions with mesocosm included as a random effect (using glmer and other functions in the lme4 package (Douglas et al. 2015) in R). However, the mesocosm term was dropped when comparing infections in the few metamorphosing survivors (using glm). Similarly, we compared the timing of epidemics by regressing the timing of mortality against treatment with mesocosm as a random effect (using lmer). We obtained test statistics using Satterthwaite's method for denominator degrees of freedom (lmerTest package; Alexandra, Per, and Rune 2017). To compare the peak viral titers among groups, we first regressed log_10_ (gene copy number) of the positive individuals against a third‐order polynomial of days postvirus introduction, for each treatment separately, to account for the rise and fall of titers over time. These models included a random intercept for each mesocosm. We then used these regressions to predict the titers over a range of days postexposure and found the peak day and peak predicted titer for each treatment. Data and complete analyses are available from the Dryad Digital Repository: https://doi.org/10.5061/dryad.w3r2280z9.

## 3 Results

Virtually all (99%) carcasses from the mesocosms with epidemics tested positive for ranavirus (2496 of 2587 tested) and just 4 of 259 (1.5%) of controls (Supplemental Results), so we are confident that the observed mortality faithfully tracks the epidemic.

**FIGURE 1:**
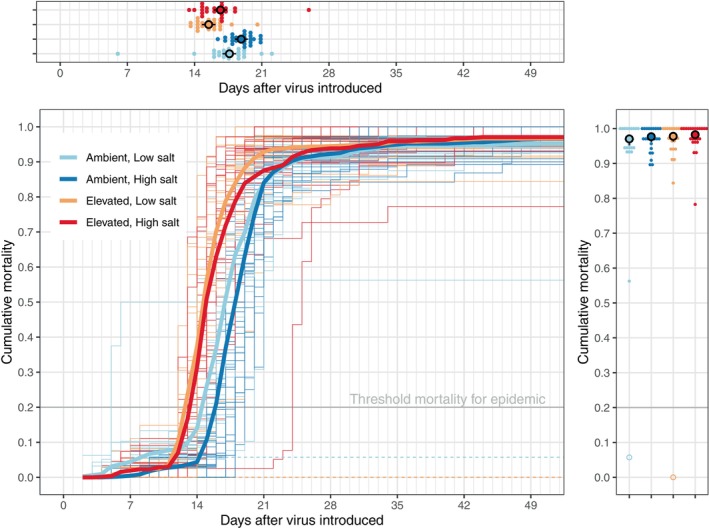
The dynamics and outcome of experimental ranavirus epidemics. The main panel shows the cumulative proportion of recovered larvae found dead over time since virus‐infected larvae were introduced into each mesocosm (thin lines; *N* = 24 per treatment) and the average across mesocosms (thick lines) within each treatment. The marginal figure on the right shows the mortality at the end of the experiment and on top the average midpoint of mortalities in each mesocosm (point). The black circles and lines are model‐derived averages and 95% CIs. Note that mortality in two mesocosms did not exceed our operational threshold for an epidemic and so were excluded from analyses (dashed lines, open circles).

Ranavirus epidemics were operationally defined as > 20% mortality to distinguish background mortality typical of mesocosm studies from the severe mortality of ranavirus epidemics in mesocosms (Reeve et al. 2013; Gallagher et al. 2019). Such epidemics occurred in all but two virus‐exposed mesocosms (Figure 1)—one in the low‐salt, ambient temperature treatment and one in the low‐salt, elevated temperature treatment—both of which showed little evidence of ranavirus spread (Figure S2; Brunner et al. 2024). While these two mesocosms that avoided epidemics were in the low‐salt treatment, this was well within typical binomial sampling variation (*β*_salinity_ = 18.43 ± 4219.34, *z* = 0.004, *p* = 0.997). Thus, epidemics were no more likely to occur in high‐salt or elevated‐temperature treatments. These treatments did not affect the severity of the resulting epidemics either (Figure 1; *β*_salinity_ = 0.24 ± 0.29, *z* = 0.83, *p* = 0.405; *β*_temperature_ = 0.29 ± 0.29, *z* = 1.02, *p* = 0.310; Table S1). Overall, the epidemics were highly lethal; the average cumulative proportion of tadpoles that died during the epidemics ranged from 0.970 to 0.982. Environmental conditions did, however, influence the timing of epidemics (Figure 1). The midpoint of epidemics in elevated‐temperature conditions occurred 2.16 ± 0.41 days postintroduction (PI) earlier, on average, than those in ambient temperatures (*t* = −5.22, df = 83.95, *p* < 0.001; Table S2), perhaps because of increased rates of transmission at higher temperatures or shorter times to death (Brand et al. 2016). High salinity instead delayed epidemics by 1.22 ± 0.41 days (*t* = −2.95, df = 83.92, *p* = 0.004).

**FIGURE 2:**
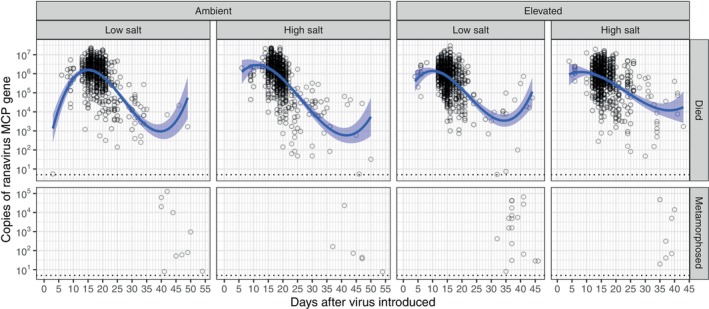
The intensity of viral infections through the experimental ranavirus epidemics. Points are the viral titer (copies of the ranavirus major capsid protein gene) in internal tissues (liver, kidney, and gastrointestinal tract as available) dissected out of animals that died (top panels) or metamorphosed (bottom panels) during the experiment. The blue lines are best‐fit third‐order polynomial regressions of log_10_(titer) against days postintroduction.

The intensity of the ranavirus infections in carcasses varied considerably over the course of the epidemics (Figure 2). They generally increased over the first 1–2 weeks PI, peaking during the primary pulse of mortality, and then declined afterward (Figure 2). The predicted peak in intensity occurred at least 2 days earlier in the elevated temperature treatments (9.65 and 9.46 days PI in the low‐ and high‐salinity treatments, respectively) compared to the ambient temperature treatment (14.9 and 11.4 days PI in the low‐ and high‐salinity treatments, respectively), suggesting viral replication rates in vivo increase with temperature. The predicted peak intensities, however, were similar across the treatments (range of 10^6.21^–10^6.43^ copies).

The prevalence of infection among the 93 metamorphosing animals that escaped epidemic mortality was roughly 60% (40 of 66 tested) and did not differ significantly among treatments (*β*_salinity_ = −0.69 ± 0.54, *z* = 1.29, *p* = 0.196; *β*_temperature_ = 0.81 ± 0.53, *z* = 1.54, *p* = 0.123; Table S3).

## 4 Discussion

While environmental stressors have frequently been shown to increase the susceptibility of individual vertebrate hosts (Dhabhar 2014; Glaser and Kiecolt‐Glaser 2005; Hall et al. 2020; Rollins‐Smith 2017), our study is unique in testing the population‐level consequences of such stress‐induced susceptibility directly, free of other causal influences (e.g., changes in host densities, exposure, etc.). Counter to our expectations, elevated salinity and temperature—two important environmental factors with known impacts on ranavirus infections and the physiology of individual animals (Brand et al. 2016; Brunner et al. 2015; Hall et al. 2020)—exerted remarkably little influence, individually or in combination, on the probability, dynamics, and outcome of replicate, experimentally initiated ranavirus epidemics (Figure 1). Both the probability of an epidemic occurring and the overall mortality these epidemics created were similar among all treatments (Figure 1). Epidemics in the elevated temperature treatment did, as expected, proceed somewhat faster—a few days earlier than in other treatments—although they did not notably shorten the duration of the epidemics (Figure 1). Elevated salinity *slowed* epidemics, delaying the midpoint of mortality by a day on average, rather than accelerating them as we might expect. These differences, however, were small relative to the variation among replicate epidemics. Overall, the lack of large differences among treatments, especially in terms of the probability and severity of epidemics, is surprising in light of prior work showing that the intensity and virulence of individual ranavirus infections are higher in larvae raised in high‐salinity conditions (Hall et al. 2020).

The absence of strong effects of these environmental manipulations is not due to small sample sizes or a large amount of variability in epidemic dynamics. We had substantial replication (*n* = 24 mesocosms/treatment exposed to ranavirus) and replicate epidemics proceeded along very similar trajectories (Figure 1). Moreover, the experimental treatments were designed to elicit large effects. Similar levels of elevated salinity have been shown to substantially decrease embryonic and larval survival (Karraker, Gibbs, and Vonesh 2015), depress larval growth and activity (Hall et al. 2017), and increase the intensity of and odds of dying from ranavirus infections (Hall et al. 2020) in wood frogs. Similarly, a 3°C increase in temperature accelerates growth and development, which we observed, increases metabolic rates (Watkins and Vraspir 2006), and, most directly relevant to our study, increases the speed and odds of dying from ranavirus infections (Brand et al. 2016). In short, these were well‐replicated, strong manipulations of important environmental conditions.

There are several possible explanations for individual‐level effects not manifesting as population‐level effects. First, environmental stressors like increased salinity can have multiple, opposing consequences for infections, which might effectively cancel each other. For instance, salinity stress has been shown to slow development rate (Hall et al. 2017), and ranavirus infections appear to increase more slowly in less developed tadpoles (Warne, Crespi, and Brunner 2011), which may explain the slightly delayed onset of mass mortality in high‐salinity mesocosms. Alternatively, salinity stress has also been shown to cause more intense ranavirus infections that lead to more viral shedding, which should increase transmission, but also to shorter infectious periods due to faster mortality (Hall et al. 2020), which should decrease transmission, at least in this study, where carcasses were removed. Perhaps these countervailing influences cancel each other. Or perhaps conditions in carefully controlled laboratory experiments, as important as they are for understanding causal relationships and mechanisms, exaggerate the influence of environmental variables on host–pathogen relationships, as they do for other interspecific interactions among anurans (Skelly 2002; Skelly and Kiesecker 2001).

Our study was unique in scale—moving from single animals in small containers or aquaria in the lab to small populations experiencing natural diurnal light and temperature cycles. However, our mesocosms were necessarily artificial, particularly in their small size, lack of habitat complexity, and absence of other community members such as predators. These simplified conditions might magnify contact rates and pathogen transmission (Brunner et al. 2017; Tompros et al. 2021). Indeed, the epidemics we observed were fast relative to what is often (partially) observed in nature (Hall et al. 2018), which might have overwhelmed the effects of our treatments. It is thus possible that elevated salinity and temperatures would have stronger effects, at least on transmission rates, in larger, more complex natural systems where transmission is presumably slower. Indeed, the two replicates without epidemics were in the low‐salinity treatment. We thus cannot exclude experimental artifacts dominating our results. However, there is a lesson here in that the somewhat subtle effects of individual differences in susceptibility can be overwhelmed by other forces that affect epidemics (e.g., high transmission rates).

Prior research in mesocosms found that ranavirus transmission is shaped by heterogeneity in susceptibility to infection—the likelihood of being infected given a contact—more than by contact rates (Brunner et al. 2017). The pattern of infection intensity we observed in carcasses over the course of the epidemic is also consistent with the hypothesis that there was a large amount of heterogeneity in infection suppression among individual larvae, regardless of treatment. Most individuals that died early in the epidemics (≤ 14 days PI) had high viral titers (~10^6^ copies), suggesting low resistance to viral replication, but others were able to reduce or keep virus titers to levels several orders of magnitude lower and survive for weeks longer. Moreover, although rare, some infected individuals survived metamorphosis (Figure 1), a large fraction of which retained their infections (Figure 2 and Figure S2), as has been observed in prior studies (Warne, Crespi, and Brunner 2011). Unfortunately, we could not determine which factors were associated with longer survival (e.g., body size or stage at infection, fat stores, and immunity), but these apparently more resistant, surviving metamorphs are likely important. They are the ones that are likely to escape from epidemics, even highly lethal epidemics, such as we generated, with sublethal infections and become reservoirs of ranavirus infection in the network of vernal ponds (Crespi et al. 2015).

Extrapolating individual‐level outcomes of host–parasite interactions to population‐level outcomes are necessary and even warranted in disease ecology (Ebert, Lipsitch, and Mangin 2000); indeed, epidemic models are built on this sort of scaling. Our study serves as a caution against assuming that individual‐level effects—even well‐studied effects of important environmental stressors—necessarily scale simply to population‐level outcomes. They may have countervailing influences that cancel out or simply be overwhelmed by other factors. This caution is especially important as scientists collectively strive to understand the consequences of our growing influence on the natural world.

## Author Contributions

**Jesse L. Brunner:** conceptualization (equal), formal analysis (lead), funding acquisition (lead), methodology (supporting), project administration (supporting), visualization (equal), writing – original draft (lead), writing – review and editing (equal). **Nicole C. Dahrouge:** formal analysis (equal), investigation (lead), methodology (equal), visualization (equal), writing – original draft (equal), writing – review and editing (supporting). **Erica J. Crespi:** conceptualization (equal), funding acquisition (equal), methodology (equal), writing – original draft (supporting), writing – review and editing (equal). **Tracy A. G. Rittenhouse:** conceptualization (equal), funding acquisition (equal), methodology (equal), project administration (lead), supervision (lead), writing – original draft (supporting), writing – review and editing (equal).

## Conflicts of Interest

The authors declare no conflicts of interest.

## Supporting information


Data S1.

## Data Availability

All data have been archived and are publicly available on the Dryad Digital Repository: https://doi.org/10.5061/dryad.6wwpzgn5q and the code at https://doi.org/10.5281/zenodo.10480144.
