# Supplementary material for: Testing the Population‐Level Effects of Stress‐Induced Susceptibility in the Ranavirus–Wood Frog System
Source: Ecol Evol. 2025 Feb 19;15(2):e70728. doi: 10.1002/ece3.70728 (PMC11836900; doi:10.1002/ece3.70728)
Supplement: Supplementary file 1 — Data S1. [file ECE3-15-e70728-s001.docx]

Supplemental information for:

Testing the population-level effects of stress-induced susceptibility in the ranavirus-wood frog system

Jesse L. Brunner, Nicole C. Dahrouge, Erica J. Crespi, Tracy A.G. Rittenhouse

Corresponding author: [jesse.brunner@wsu.edu](mailto:jesse.brunner@wsu.edu)

Supplemental results

There was little mortality in control mesocosms (*45*), nearly always less than 10%, and later than the epidemics when tadpoles were beginning to metamorphose (Fig. S1). Epidemics were operationally defined as >20% mortality following ranavirus introduction. In the two mesocosms that did not exceed this amount of mortality ranavirus was found in 0 of 24 animals tested in one and 1 of 26 in the other, suggesting ranavirus infections did not spread. The vast majority of recovered animals were screened for ranavirus DNA with quantitative real time PCR (Fig. S2). Those that were not screened were either lost or skeletonized and thus lacked tissue from which to extract DNA. In contrast, 98% of the 2587 individuals screened from the remaining 94 ranavirus-exposed tanks tested positive, including 99% of the 2521 individuals that died (Fig. S2). We are thus confident the epidemic mortality was caused by ranavirus infections.

All but four of the 259 (98.5%) control animals tested for ranavirus were negative (Fig. S2). Those four positive animals came from all four treatments. Three who were euthanized after metamorphosis had low copy numbers (<15), which may be consistent with contamination in the laboratory, but one in the low salinity + elevated temperature treatment that died had a relatively high (3974) number of copies.

Treatment did not have a statistically significant effect on the odds of animals dying in the virus-exposed tanks (Table S1). The timing of epidemics, however, was significantly affected by both salinity and temperature treatments (Table S2). The proportion of surviving animals that were positive for ranavirus was not significantly different among treatments (Table S3).

**Table S1**: Results of a logistic regression comparing mortality among ranavirus-exposed tanks by treatment with tank identity included as a random effect. The R code was: glmer(cbind(Dead,Live) ~ Salinity + Temperature + (1|Tank), family="binomial")

|  | Estimate | SE | *z* | *P* |
| --- | --- | --- | --- | --- |
| Intercept | 3.731 | 0.262 | 14.251 | < 0.001 |
| Low salt | -0.241 | 0.289 | -0.833 | 0.405 |
| Elevated temp. | 0.295 | 0.290 | 1.016 | 0.310 |

**Table S2**: Results of a regression of the timing of mortality (i.e., days virus introduction) against treatment with tank identity included as a random effect. The r code was:

lmer(DaysPI ~ Salinity + Temperature + (1|Tank))

|  | Estimate | SE | *t* | *P* |
| --- | --- | --- | --- | --- |
| Intercept | 18.856 | 0.356 | 52.937 | < 0.001 |
| Low salt | -1.225 | 0.415 | -2.954 | 0.004 |
| Elevated temp. | -2.164 | 0.415 | -5.221 | < 0.001 |

**Table S3**: Results of a logistic regression of infection status of individuals that survived ranavirus-induced epidemic mortality (i.e., metamorphosed) by treatment. Note: tank identity is not included in this model. The R code was:

glm(cbind(Positive,I(N-Positive))~Salinity+Temperature, family="binomial")

|  | Estimate | SE | *z* | *P* |
| --- | --- | --- | --- | --- |
| Intercept | -0.406 | 0.516 | -0.786 | 0.432 |
| Low salt | 0.693 | 0.537 | 1.292 | 0.196 |
| Elevated temp. | 0.811 | 0.526 | 1.540 | 0.123 |

Fig. S1.

The cumulative proportion of animals found dead, as opposed to euthanized as metamorphs, in ranavirus-exposed and control mesocosms over time in individual mesocosms (thin lines) and averaged across mesocosms (thick lines). The horizontal dotted line represents threshold amount of mortality we used to operationally define epidemics. Note that mortality in the control mesocosms occurred later than that in the epidemics, when tadpoles were metamorphosing.

Fig. S2.

The PCR status of internal tissues (liver, kidney, and gastrointestinal tract as available) dissected from wood frogs found dead (Died) or euthanized as metamorphs (Meta) following the introduction of ranavirus-infected tadpoles (Virus) or uninfected control tadpoles (Control) in the ambient and elevated temperature and low and high salinity treatments. Data from the two virus-exposed mesocosms that did not experience epidemics is not included in this figure so as to allow a clearer comparison in the status of survivors of epidemics. Note that ranavirus-related mortality tended to occur well before tadpoles began to metamorphose..
